# Barriers and facilitators of participation in syphilis vaccine trials: a qualitative analysis to inform trial design and community engagement in the United States

**DOI:** 10.1080/26410397.2025.2473199

**Published:** 2025-03-07

**Authors:** Suzanne Day, Asia Carter, Anna Lloyd, Arlene C. Seña, Justin D. Radolf, Joseph D. Tucker

**Affiliations:** a Research Assistant Professor, Department of Medicine, Division of Infectious Diseases, University of North Carolina at Chapel Hill, 130 Mason Farm Road, Chapel Hill, NC 27514, USA.; b Graduate student, Department of Health Behavior, Gillings School of Global Public Health, University of North Carolina at Chapel Hill, Chapel Hill, NC, USA; c Undergraduate student, Department of Biology, University of North Carolina at Chapel Hill, Chapel Hill, NC, USA; d Professor, Department of Medicine, Division of Infectious Diseases, University of North Carolina at Chapel Hill, Chapel Hill, NC, USA; e Professor, and Director of Research for the Department of Medicine, Departments of Medicine and Pediatrics, UConn Health, Farmington, CT, USA; f Professor, Clinical Research Department, Faculty of Infectious and Tropical Diseases, London School of Hygiene and Tropical Medicine, London, UK

**Keywords:** syphilis, vaccine research, clinical trial participation, key populations, community engagement

## Abstract

Amidst resurging syphilis infection rates, increasing efforts are being made towards development of a syphilis vaccine. This study aims to identify barriers and facilitators of syphilis vaccine trial participation among priority groups for early phase studies. We conducted interviews with English-speaking individuals ≥18 years old recruited from an infectious disease clinic, a sexually transmitted infection (STI) testing site, an online research bulletin board, and HIV community advisory boards in North Carolina from April 2021–June 2022. Eligibility criteria included STI diagnosis within 12 months, people living with HIV (PLWH), men who have sex with men, or persons engaged in transactional sex. The interview guide examined views on syphilis vaccines, trial participation, and community engagement. Interviews were transcribed verbatim, coded, and analysed for emergent themes using a social ecological model. Thirty individuals were interviewed, including eight (27%) women, 13 (43%) Black/African American individuals, and 19 (63%) PLWH. While 19 (63%) interviewees were interested in syphilis vaccine trial participation, 10 (33%) noted participation would depend on trial parameters; one person expressed no interest. Trial participation barriers included physical risks, time commitments, and concerns related to mistrust and mistreatment. Facilitators included advancing science, syphilis prevention, and trusting the researchers. Interviewees emphasized the importance of community involvement to inform vaccine trials, particularly amidst the lingering shadow of the Tuskegee Syphilis Study. While priority groups thus expressed interest in syphilis vaccine trial participation, tailored community engagement will be essential prior to clinical trials. Additional mixed methods research is urgently needed.

## Plain language summary

As syphilis infection rates are on the rise, efforts are being made to develop a syphilis vaccine. We wanted to learn what key groups think about participating in syphilis vaccine trials. From April 2021 to June 2022, we interviewed English-speaking adults in North Carolina. We recruited people via infectious disease clinics, testing sites, an online bulletin board, and HIV community advisory groups. People could take part if they (1) had experienced a sexually transmitted infection in the past year, (2) were living with HIV (3) were men who have sex with men, or (4) engaged in transactional sex

We asked people what would encourage or discourage them to take part in syphilis vaccine trials. We also asked how they thought communities could be engaged on the topic of syphilis vaccine research. 19 out of 30 individuals expressed interest in joining syphilis vaccine trials. Ten noted that participation would depend upon trial specifics, and one person would not participate in trials at all. Participants said concerns about vaccine safety could be discouraging. Other concerns were time commitments, and how researchers would treat trial participants. Personal motivations like advancing science and preventing syphilis would encourage them to take part. They also mentioned that trust in researchers would be important to encourage people to join trials. Participants recommended improving community involvement in trial planning. Researchers should draw lessons from historical injustices like the Tuskegee Syphilis Study. This study highlights the importance of conducting tailored community engagement before large-scale syphilis vaccine trials.

## Résumé

Dans un contexte de résurgence des taux d’infection syphilitique, des efforts croissants sont déployés pour mettre au point un vaccin contre la syphilis. Cette étude vise à identifier les obstacles et les facilitateurs de la participation aux essais des vaccins contre la syphilis parmi les groupes prioritaires pour les études de la phase précoce. Nous avons mené des entretiens avec des anglophones âgés de plus de 18 ans recrutés dans un centre de traitement des maladies infectieuses, un site de dépistage des infections sexuellement transmissibles (IST), un panneau d’affichage de recherche en ligne et des conseils consultatifs communautaires sur le VIH en Caroline du Nord d’avril 2021 à juin 2022. Les critères d’éligibilité incluaient les personnes ayant eu un diagnostic d’IST dans les 12 mois précédents, les personnes vivant avec le VIH, les hommes ayant des rapports sexuels avec des hommes ou les personnes pratiquant des rapports sexuels transactionnels. Le guide d’entretien examinait les points de vue sur les vaccins contre la syphilis, la participation aux essais et l’engagement communautaire. Les entretiens ont été retranscrits mot pour mot, codés et analysés pour y rechercher des thèmes émergents à l’aide d’un modèle écologique social. Trente personnes ont été interrogées, dont huit (27%) femmes, 13 (43%) Noirs/Africains-Américains et 19 (63%) personnes vivant avec le VIH. Alors que 19 (63%) personnes interrogées étaient intéressées par une participation à l’essai du vaccin contre la syphilis, 10 (33%) ont indiqué que leur participation dépendrait des paramètres de l’essai; une personne n’a manifesté aucun intérêt. Les obstacles à la participation à l’essai comprenaient les risques physiques, les engagements en termes de temps et les préoccupations relatives à la méfiance et aux mauvais traitements. Les facilitateurs incluaient l’avancement de la science, la prévention de la syphilis et la confiance envers les chercheurs. Les personnes interrogées ont souligné l’importance de la participation communautaire pour guider les essais de vaccins, en particulier dans le contexte de l’ombre persistante de l’étude de Tuskegee sur la syphilis. Si les groupes prioritaires ont ainsi exprimé leur intérêt pour une participation aux essais du vaccin contre la syphilis, un engagement communautaire adapté sera essentiel avant les essais cliniques. Des recherches complémentaires avec des méthodes mixtes sont nécessaires de toute urgence.

## Resumen

En respuesta al resurgimiento de las tasas de infección por la bacteria que causa la sífilis, se están realizando cada vez más esfuerzos por crear una vacuna contra la sífilis. Este estudio pretende identificar las barreras y los facilitadores de la participación en ensayos clínicos de la vacuna contra la sífilis entre grupos prioritarios para estudios de fase temprana. Entre abril de 2021 y junio de 2022, realizamos entrevistas con personas anglófonas ≥ 18 años reclutadas de una clínica de enfermedades infecciosas, un sitio de pruebas de infecciones de transmisión sexual (ITS), un tablón de anuncios de investigación en línea y consejos asesores comunitarios sobre VIH en Carolina del Norte. Los criterios de elegibilidad eran: diagnóstico de ITS en un plazo de 12 meses, personas que viven con VIH (PVV), hombres que tienen sexo con hombres (HSH), o personas que participan en sexo transaccional. La guía de entrevistas examinó los puntos de vista sobre las vacunas contra la sífilis, la participación en ensayos clínicos y la participación comunitaria. Las entrevistas fueron transcritas palabra por palabra, codificadas y analizadas para identificar temas emergentes utilizando un modelo ecológico social. Se entrevistaron 30 personas, entre ellas 8 (27%) mujeres, 13 (43%) personas negras/afroamericanas y 19 (63%) PVV. Si bien 19 (63%) de las personas entrevistadas estaban interesadas en participar en un ensayo clínico de la vacuna contra la sífilis, 10 (33%) indicaron que su participación dependería de los parámetros del ensayo; una persona expresó no tener interés. Las barreras a la participación en el ensayo eran: riesgos físicos, compromisos de tiempo y preocupaciones relacionadas con la desconfianza y el maltrato. Los facilitadores eran: el avance de la ciencia, la prevención de sífilis y la confianza en los investigadores. Las personas entrevistadas hicieron hincapié en la importancia de la participación comunitaria para informar los ensayos de vacunas, en particular debido a la sombra persistente del Estudio de Sífilis en Tuskegee. Aunque los grupos prioritarios expresaron interés en participar en ensayos clínicos de la vacuna contra la sífilis, la participación comunitaria personalizada será esencial antes de realizar los ensayos clínicos. Se necesitan urgentemente investigaciones de métodos mixtos adicionales.

## Introduction

Syphilis is a global public health problem, endemic in many low- and middle-income countries (LMICs) and resurgent in high-income countries.^1^ In 2023, the rate of syphilis infection in the United States (US) was 61.3 per 100,000, with the number of cases of all stages of syphilis increasing 1% from 2022, and syphilis of an unknown duration and late-stage increasing 12.8% in the same period.^2^ While prevention, early detection, and treatment have been core pillars of the public health response to this sexually transmitted infection (STI), vaccine development is urgently needed as a parallel strategy for syphilis elimination.^3^ As basic research advances towards identification of viable vaccine candidates,^4^ there is a need to understand social and ethical factors that may influence participation in syphilis vaccine trials.

The history of syphilis research is fraught with some of medicine’s most well-known ethical violations. Beyond the infamous Tuskegee syphilis study in the US,^5^ unethical syphilis research has also been conducted in France,^6^ the United Kingdom,^7^ and China.^8–10^ For example, in 2010 the public learned of the syphilis experiments in Guatemala in which US government scientists deliberately inoculated subjects with *Treponema pallidum*, the causative agent, in order to study its transmission.^11^ The Sing Sing prison experiments also involved intentionally infecting incarcerated people with syphilis in order to determine at which stage individuals with prior syphilis infection develop immunity to reinfection.^12^ The complex history of syphilis research raises questions about the lasting impact that these events may have on willingness to participate in clinical STI research,^13^ particularly among minoritised communities.^14^ Lingering mistrust in research due to the Tuskegee syphilis studies has been shown to be a significant barrier to participation in medical research among African Americans,^13^ and trust-building has been noted to be of central importance for STI research participation among Black sexual minority men.^15^

Syphilis continues to be highly stigmatised in its association with sexual behaviour,^16^ as well as in its disproportionate impact on populations along racial/ethnic^17,18^ and class lines.^19^ In the US, groups experiencing disproportionate rates of syphilis include men who have sex with men (MSM) and people living with HIV (PLWH).^20^ Rates of syphilis infection have also risen among heterosexual women, while delays in prenatal care and treatment among pregnant women have contributed to alarming increases in congenital syphilis.^21^ As efforts to develop a syphilis vaccine progress,^4,22^ understanding perspectives regarding vaccine development among these priority populations is of central importance in helping to anticipate organisational, social, and ethical issues that may impact participation in vaccine clinical trials. Such understanding via consultation with impacted marginalised populations is essential for ensuring clinical trials are designed in ways that protect human rights.^23^ To address these needs, the goals of this study were to examine barriers and facilitators of participation in syphilis vaccine trials among priority groups located in the southeastern US and to identify key population perspectives to inform participatory community engagement strategies for syphilis vaccine development.

## Materials and methods

### Data collection

Our study team was comprised of infectious disease researchers specialising in syphilis and working towards syphilis vaccine development (JDT, ACS, JDR), social scientists specialising in qualitative and participatory approaches to health research (SD, JDT), and student research assistants in the fields of public health (AC) and biology (AL). This qualitative study is structured according to a thematic analysis framework,^24^ an approach for rich description of dataset patterns that emerge in response to an organising research question: what are the barriers to and facilitators of syphilis vaccine trial participation? We used semi-structured interviews to investigate the perspectives of individuals who would most likely be eligible for participation in early-phase syphilis vaccine clinical trials due to belonging to groups that experience increased rates of syphilis infection. Eligibility criteria included individuals who were age 18 years or older who had either been diagnosed with any STI in the past 12 months or who self-reported being a member of the following key populations: people living with HIV (PLWH), men who have sex with men (MSM), or people who engage in transactional sex.

Interview participants were recruited from 7 April 2021 to 8 June 2022 using study flyers at a university-affiliated infectious disease clinic (serving approximately 2300 patients), a public health STI clinic (2795 patients served in 2023), and HIV community advisory boards located in Chapel Hill, Durham, and Raleigh, North Carolina. We also posted the opportunity for study participation on a university-affiliated online research bulletin board. Individuals interested in participating self-referred to the study coordinator who screened for eligibility over the phone and scheduled eligible individuals for an interview. Interviews were conducted one-to-one in English over Zoom videoconference^25^ using a secure link provided to participants in advance. Interview participants were also provided with the study’s informed consent form in advance of the interview. The interviewer also read the informed consent form aloud to each participant prior to beginning any study activities. Participants provided verbal consent to be interviewed in lieu of written consent, which was then documented by the interviewer. This study received ethical review and approval on February 11, 2021 from the University of North Carolina at Chapel Hill (UNC) Institutional Review Board, protocol number Protocol # 20-3701. Our results are reported using the Standards for Reporting Qualitative Research (SRQR) guidelines.^26^

**Figure 1.**
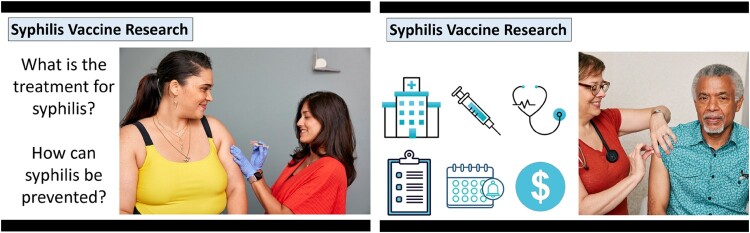
Two screenshots of the narrated animation shown in each interview

Interviews were conducted by a researcher with expertise in qualitative methodology (SD) using an interview guide (see Supplemental Material 1) designed to explore in approximately one hour an array of topics relevant to informing the design of early-phase syphilis vaccine clinical trials and related community engagement efforts. These topics included interviewees’ views on the idea of a syphilis vaccine, trial participation barriers/facilitators, and community involvement in vaccine research. All interviews were audio recorded. To make the topic of syphilis vaccine clinical trials easier to understand, interviewees were shown a plain-language 5-minute narrated video designed by the study team (Figure 1; viewable online at https://vimeo.com/987696938) to provide more information about what syphilis is and how a vaccine would be developed. The video (which was shown midway through the interview) also provided interviewees with a better understanding of what participation in an early-phase (i.e. phase 1, non-randomised) vaccine trial would involve (e.g. receiving an injection of the vaccine to be tested, monitoring for adverse effects, repeat clinic visits). Interviewees watched the video on Zoom with the interviewer who would pause to answer questions as needed.

After each interview, demographic data were collected from interviewees using an online survey, the link to which was provided via email. Participants received a $35 gift card as compensation for their time.

### Data analysis

Interviews were transcribed verbatim by a professional transcription service. Two coders iteratively developed a codebook for export to MAXQDA coding software,^27^ starting with a deductive list of parent/child codes informed by a social ecological model^28^ of individual-, interpersonal-, institutional-, community-, and policy-level factors that could serve as barriers to or facilitators of syphilis vaccine trial participation. We chose this model because it provides actionable levels for potential interventions to address trial participation and has been used previously to address barriers to clinical trial enrolment and retention in minority communities.^29^ The two coders collaboratively revised the initial list of deductive codes in three subsequent rounds of revisions to add inductive subcodes identified through close reading of a set of three interview transcripts. The two coders proceeded to test-code a set of five interview transcripts using the codebook and compared coding to determine fidelity between coders, with subsequent adjustments made to finalise the codebook. The two coders then divided the transcripts for independent coding using the finalised codebook. Coders then exchanged coded sets of transcripts with each other to evaluate fidelity in coding with the goal of consensus, with all discrepancies resolved through review and discussion to reach agreement. The finalised set of all coded data was then analysed for emergent themes and organised using a social ecological model to identify key themes pertaining to participation in a syphilis vaccine trial. We further analysed coded data to explore participants’ views on the idea of a syphilis vaccine, interest in syphilis vaccine trial participation, as well as perceptions regarding community involvement in syphilis vaccine research.

## Results

We interviewed 30 people. Interviewees’ demographic information is summarised in Table 1.

**Table 1.** Demographic characteristics of interviewees, *N* = 30, in the United States, 2021–2022

Interview participant characteristics		*n* (%)
**Age (years)**	18–24	5 (17%)
	25–34	5 (17%)
	35–44	4 (13%)
	45–54	5 (17%)
	55–64	7 (23%)
	65–74	4 (13%)
**Gender**	Cisgender Man	18 (60%)
	Cisgender Woman	8 (27%)
	Transgender/Non-Binary	4 (13%)
Race/Ethnicity*	Black, African American, Afro-Caribbean or of African descent	13 (43%)
	White or Caucasian, European descent	13 (43%)
	Multi-racial/ethnic	3 (10%)
**Education completed**	Did not complete high school	1 (3%)
	High school diploma or GED	5 (17%)
	Some college, no degree	4 (13%)
	Associate’s degree or Bachelor’s degree	14 (47%)
	Master’s degree	6 (20%)
HIV status**	Not living with HIV	8 (27%)
	Living with HIV	19 (63%)
Ever (i.e. at any time) had an STI test (non-HIV)***	Yes	27 (90%)
	No	1 (3%)
**Ever (i.e. at any time) had positive STI (non-HIV) test result at any time**	Yes	19 (63%)
	No	8 (27%)
	N/A – never tested/not sure if ever tested/would rather not say	3 (10%)
**# of partners in past month**	None	12 (40%)
	One	7 (23%)
	Two or more	11 (37%)
**Ever (i.e. at any time) participated in clinical research**	Yes	22 (73%)
	No	7 (23%)
	Don’t know	1 (3%)

* For the question of race/ethnicity, one participant indicated “would rather not say” (3%).

** For the question of HIV status, one participant indicated “don’t know” (3%), one participant indicated “N/A” (never tested) (3%), and one participant indicated “would rather not say” (3%).

*** For the question of ever having had an STI test (non-HIV), one participant indicated “would rather not say” (3%), and one participant indicated “I don’t know” (3%).

Our sample included individuals from populations disproportionately impacted by syphilis (e.g. people living with HIV) and likely to be of priority in syphilis vaccine research (e.g. people with previous STI infection). Of the 30 participants, 14 (47%) were between the ages of 18–44 years old, with just over half (16; 53%) 45+ years. Most participants identified as cisgender men (18; 60%), although eight (27%) identified as cisgender women and four (13%) as a gender minority (transgender or non-binary). Participants consisted of an equal number of white/Caucasian and black/African American (or of the African diaspora) at 13 (43%) participants, respectively, with an additional three (10%) participants identifying as multi-racial/ethnic. Nineteen participants (63%) were people living with HIV (PLWH). Participants had high levels of experience with testing for STIs, with 27 (90%) reporting that they had ever (i.e. at any point in time) been tested for an STI other than HIV and 19 (63%) reporting having ever had a positive STI test. Most reported having at least one sexual partner in the past month, with seven (23%) reporting one partner and 11 (37%) reporting two or more partners in the past month. Most (22; 73%) reported past participation in clinical research studies.

### Perceptions of a syphilis vaccine

When asked what they thought about the idea of a syphilis vaccine, all participants reported that this would be an important and helpful innovation. We also found high levels of interest in vaccination among participants, with 25 (83%) reporting that they would be interested in being vaccinated if a syphilis vaccine were available. Three participants (10%) reported they would not be interested, explaining that the vaccine would not be relevant to them due to not currently being engaged in sexual activity, and two (7%) were undecided.

When asked to explain who they thought would be most in need of a syphilis vaccine, individual participants named a wide variety of groups perceived to be at greater risk, including young people, transgender women, and MSM. However, participants also named broader groups based on the need for widespread protection from syphilis: people with multiple sexual partners, anyone who was sexually active, or even “everybody as a whole” [P22] should be vaccinated for as broad protection as possible.

### Interest in syphilis vaccine trial participation

After watching the video that explained basic procedures that would be involved in a syphilis vaccine trial, interviewees were asked to consider whether they would hypothetically be interested in participating in such a trial – notably framed as an early-phase, first-in-humans study. Of the 30 interviewees, 19 (63%) confirmed that they would be interested in trial participation, while 10 (33%) explained that their interest in participation would be contingent on a variety of factors (examined below as barriers to and facilitators of trial participation). Only one interviewee expressed no interest in participation. Regardless of an interviewee’s level of interest, the interviewer continued to probe for concerns or expectations regarding potential trial participation. Our thematic analysis of responses to questions about views on trial participation revealed a range of factors that served as barriers to or facilitators of vaccine trial participation. These factors can be organised according to a social ecological model, as summarised in Table 2 and examined in-depth.

**Table 2.** Summary of the thematic analysis of factors posing barriers to and facilitators of syphilis vaccine trial participation, organized according to a social ecological model

Factor Level	Barriers	Facilitators
**Individual**	Fear of physical harm - Side effects/injury - Syphilis exposure	Benefiting from the vaccine/trial - Being vaccinated - Obtaining medical care Lower perceived risk
**Interpersonal**	Treated like a lab-rat	Trustworthy researchers
**Institutional**	Time involved in trial participation	Trust in the research design/organization
**Community**	Community mistrust	Advancing research to end suffering/improve public health
**Policy**	Impact of the COVID-19 pandemic	Compensation Regulation of clinical trials

### Individual-level barrier: fear of physical harm

The most prevalent concern among interviewees was the fear of physical harm associated with being a vaccine trial participant, both in terms of the risks associated with vaccine administration as well as potential syphilis exposure. Concerns about physical harm from the vaccine manifested in a variety of ways; foremost among these were vaccine adverse events, or a “negative reaction” [P13]. As one participant noted, “*I will kinda be worried about how it would affect my body, what it would do to me. That would be the only thing, I think. Would it make me sick to take this medicine and stuff?*” [P27]. The duration of side effects was also an issue; one participant, a caregiver for a relative, noted that she would be concerned with “*how long the side effects would be. Is it somethin’ that you’d carry with you forever? Cause some side effects, they come and go forever, it seem like, and then some are just for a day or two, or maybe a week, a few days, and then it’ll go away.*” [P20]. The physical risks of being a trial participant also were mentioned by multiple interviewees in terms of “*how [the vaccine is] gonna affect the organs*” [P22]; for example, “*things like my heart. Things like my kidneys, my brain*” [P5], or that the vaccine “*would somehow make the heart do somethin’ bad and … trigger somethin’ that would make my heart go [makes exploding sound]*” [P12]. Two interviewees further referred to mortal danger associated with trial participation given the unknowns and wide range of possible reactions, noting that there was a risk of “*many possibilities, including death, but that’s a very small*” [P13], because “*you inject it into the wrong place—I don’t know, there’s just so many things that could go wrong, you just don’t know*” [P4].

Two participants further noted that, with clinical trials, there are risks not only in terms of the unknowns as to what could go wrong but also in terms of what is potentially possible to know, given that “*there are only so many things you can measure safety-wise. There is always an under-the-radar, at least theoretical potential that there’s consequences beyond the trials. Those are super improbable, in my estimation, but I guess there’s still potential there”* [P2]. Another participant expressed a similar concern regarding the limits of what can be known in a trial setting:

*“What if I were to join this trial and it just so happened to be one thing within the vaccine that I am allergic to? That would be the wrong time to find somethin’ like that out. I think that would always be on the forefront of my mind, just any sort of adverse effects, things that I maybe didn’t plan for or maybe even this study personnel didn’t plan for.”* [P9]

Concerns about physical harm also informed the conditions under which some interviewees would be willing to participate in a syphilis vaccine trial. For example, one participant tied the risks of physical harm to the concept of compensation, noting that compensation would have to be sufficient to outweigh concerns about safety of the vaccine:

*“I’ve thought about before, what it would be like to participate in an experimental drug or vaccine. I think always what comes to my head is what if it’s not safe? What if something does happen to me? Knowing that it was tested in animals first, that’s definitely a—it makes me feel better, but I think there’s still is that small concern of what if something goes wrong? As long as you get compensated a decent amount, that might not be as big of a worry for me.”* [P14]

Concerns about physical harm similarly informed answers regarding participation in various phases of clinical trials. For example, one interviewee suggested that, in particular, concerns about unknown physical risks could be a barrier for trial participation in early-phase research:

*“I think I’d be probably less inclined to participate if the risks were unknown [i.e. in early phase studies], especially knowing that some of the complications to certain vaccines can be very severe, very unpleasant. I’m okay with possibly experiencing symptoms, things like that, or something else that I can deal with. I don’t know. If it were long-lasting damage—the possibility of long-lasting damage, I think that I would be more skeptical and might not participate.”* [P11]

It should be noted that for our one interviewee who expressed no interest in participating in a clinical trial for a syphilis vaccine, the physical risks associated with trial participation were the primary reason for lack of interest. Concerns were raised about “*somethin’ not goin’ right after I get the vaccine … side effects, serious side effects*”. [P9]

Multiple interviewees further noted concerns with the physical risks of participation in a vaccine trial related to the potential for syphilis exposure, with worries that the vaccine could potentially transmit syphilis, be ineffective in preventing syphilis, or make one more vulnerable to syphilis infection. For example, in reference to (incorrect) beliefs about the “flu shot”, one interviewee expressed concern with the vaccine potentially transmitting syphilis:

*“I think it would be scary at the same time, just for people that trying to get involved in [clinical trials] because at some point, they’re wondering, do I have to get [syphilis], or will the vaccine give it to me? Kinda like how the flu shot gives people the flu, some people are gonna think, well, if I get the vaccine for syphilis, what if the vaccine gives it to me?”* [P17]

Efficacy was also a concern, with some interviewees noting that trial participation could cause “*a false sense of security*” [P19] if the vaccine did not actually work. As one interviewee noted:

*“I’d be worried about getting syphilis [laughter] even post-vaccine without definitive information about how effective it is … relying on something to work that you’re not entirely sure does. You’re pretty sure. I feel like, if you get to that point, you’re pretty sure it’s gonna work, but you’re not certain.”* [P18]

Another interviewee drew on recollections of having heard about a previous trial for a vaccine that not only did not work but made participants more vulnerable:

*“I know that there was one vaccine that it turned out they didn’t do enough testing on. For some people that actually made them more likely to get that particular disease. They then had to pull it once they figured that out. That’s the type of thing I might think about, and would potentially be a little bit worried about it, especially when it was still in the research stages.”* [P1]

Finally, some interviewees noted that, rather than physical risks associated with the vaccine, exposure to syphilis as a part of a trial was also worrisome. As one noted, “*Well, if somebody says, we’d like you to participate in a trial to test a vaccine for syphilis, the first thing my mind’s gonna go to, is: are they gonna give me syphilis to test this vaccine?”* [P16]. Two interviewees similarly raised this question in reference to the Tuskegee syphilis studies, with one noting a concern with whether trial participants “*[will have to be] given the disease. I’m goin’ back to Tuskegee now. Will you have to have the disease to [be in] the study, to have this if you are a participant?”* [P20].

### Interpersonal-level barrier: being treated like a “lab rat”

A frequently raised concern was that researchers would focus on vaccine development at the expense of providing compassionate, person-centred care to trial participants. Several participants noted their concern about being treated “*like a lab rat, essentially just someone that you’re just testing things on”* [P9] – in other words, an object or number that is treated “*not as human as they’re supposed to be”* [P5], that exists solely to generate data for the trial and the benefit of the researchers conducting the study. One participant noted this as a feeling of being “*a rat in a cage … I’m just here so you can drain my blood”* [P16]. Another interviewee with prior medical research participation experience expressed dismay with how a risk of trial participation is that “*you feel like you’re in this box by yourself”* in being isolated as a data-generating point in the study, rather than as part of a team [P17]. Yet another participant spoke bluntly in terms of a syphilis vaccine clinical trial being a form of exploitation by researchers, specifically exploitation of poor people:

*“To be really honest with you, it’s about some rich ass cracker trying to give someone broke, money to test their crap and make more money. That’s all it is. They don’t care about people having syphilis, they care about the money they can make off of that vaccine. They use money to get human lab rats. Point blank, period, no other way around that. I mean, something that really helps people, yeah. When it comes to this, it’s mostly about dollars and cents.”* [P3]

This viewpoint was echoed by an interviewee who noted a bad previous trial participation experience in which “*I felt like [the researcher’s] motivation was getting her data, and she didn’t care about my health … that was even worse than just not participating*” [P19]. Another participant similarly noted that knowing that the clinical trial researchers were genuine in their desire to help prevent syphilis would make a difference:

*“If I feel like you’re just trying to make [me] your test rat, then, no, I probably won’t be the right person for you. If I feel like you’re actually trying to save lives and actually trying—you’re working on this vaccine, it’s not your side project, no, this is your life that you’re putting into, then, yes. That’s somebody who I want to help out at least.”* [P4]

### Institutional barrier: time involved in trial participation

The time commitment of being involved in a syphilis vaccine trial emerged as a barrier for several participants. Time could be a barrier, as in the total amount of time one would be enrolled in the trial – for example, while one participant noted that they would be willing to sign up for a syphilis vaccine trial, “*I can’t say a hundred percent sure I’m gonna be here for two years”* [P1]. But time also was noted as a barrier in terms of the number and length of appointments (*“It can get in the way of life, where you have to go to the doctor, and it’s, whatever, a three-hour thing*” [P2]), commute time to the study site (*“I’d get stuck in traffic or something”* [P24]), and overall the amount of time to complete particular trial tasks (*“Just the time commitment. It’s a bit of a logistical commitment to do the daily diaries”* [P18]). The ability to schedule around work and other commitments was noted by multiple interviewees as a factor in considering trial participation. Consider for example how one interviewee weighed trial participation against the scheduling of daily life:

*“If some people, if they say exactly how much of my time is this gonna take, that’s definitely a factor. Is it gonna be too much of my life? I have other things going on. How flexible am I going to be? Do I have the time to be able to be here as – inasmuch as I need to? The level of involvement. Definitely that.”* [P17]

### Community-level barrier: community mistrust

Some participants noted that a lack of trust in the researchers or the research itself could be a barrier to participation in syphilis vaccine trials, particularly among minoritised and marginalised populations. For example, one participant noted it would be particularly important for clinical trial researchers to involve “*minorities just in general just with the history of medical research and minorities. I would love for them to be included, but, at the same time, I completely understand why there’s so much mistrust with medical research”* [P9]. Others who have also had bad research experiences in the past may be reluctant to participate, for “*if they’ve been burned by somebody else, you’re not gonna have much of a chance”* [P15] at recruiting them. Furthermore, the ethical violations of Tuskegee also featured prominently in several explanations for the impact of community mistrust on syphilis vaccine trial participation:

*“I wanna say, as a Black person, it is associated with the Tuskegee syphilis things, and it’s negative. When I think syphilis, I think, oh, those vaccine trials and how horrible it was. It is very tied to that, so that’s my knowledge of syphilis.”* [P30]

When expressing his view as to the most important populations to involve in syphilis vaccine trials, another interviewee noted that some populations may need to be “convinced” to participate due to their marginalised status and lack of trust:

*“I’m gonna go with [MSM] and my prostitutes. That would be kinda tricky ‘cause you’re gonna have to convince ‘em that—“We’re not using your name. We’re not using your face. You’re just in the study. You don’t have a name.”* [P23]

### Policy-level barrier: impact of the COVID-19 pandemic

Finally, the context of COVID-19 was drawn upon by some participants to express concerns about how the pandemic could potentially pose a barrier to vaccine trial participation. For example, one interviewee noted that the rise of the anti-vaccination movement may make things more difficult in the future for trial participation:

*“I think that’s really difficult, especially now with all of the anti-vaxx movement that’s rising up with COVID. I think it’s gonna be really difficult to start having vaccine trials in the future … because I think this pandemic has hurt public health and trust in science a lot.”* [P20]

### Individual-level facilitator: benefiting from the vaccine/trial

A prominent theme as a facilitator of vaccine trial participation was the opportunity to personally benefit from being involved in future trials, both in terms of being protected from syphilis should the vaccine be effective and also regarding the medical care a trial participant would receive. As one participant noted, “*let’s say if I get syphilis, I already got the vaccine inside of me, so therefore I can’t get it. It helps me. It helps my health”* [P25]. Multiple interviewees with a history of prior syphilis noted that protection from syphilis would save trial participants from having to “*go get that damn shot at the health department”* [P3] to treat syphilis infection in the future; as one interviewee who had been treated multiple times for syphilis noted, he would be excited to participate in a vaccine trial because “*I do not wanna get those shots anymore, being infected. That’s one thing I do not want to have”* [P5]. For another participant, the opportunity to be vaccinated “*may give me a longer life span … ’Cause if you’re healthy, then … after the vaccine, you’ll live longer”* [P22] due to being protected from syphilis infection.

Others noted the benefits in terms of the medical care trial participants would receive – as one interviewee put it, the opportunity to “*get some freebies along the way, get some free lab work”* [P24] was particularly appealing to help with monitoring their health. Another interviewee similarly noted trial participation as a chance for more attentive monitoring of health status:

*“To me, any trial you participate on is just another way for your body to be monitored and catch if anything happens to go wrong. That’s my philosophy about trials [laughs] … that’s kind of what I – think about it as a way of getting an extra checkup to find out if anything might go wrong, you know?”* [P30]

### Individual-level facilitator: lower perceived risks

It is important to note too that, in contrast to the frequently noted risk of physical harm, some participants felt that a syphilis vaccine clinical trial would actually be a lower-risk study to join given that syphilis is treatable. As one participant put it, if the vaccine was found to be ineffective, “*what would be the worst thing [that could happen]? To get syphilis and get penicillin again? [Laughter]”* [P26]. Noting that syphilis has an effective treatment, this participant felt that the worst danger would be simply receiving treatment once again for a syphilis infection – something he had already experienced before. Another participant similarly noted that, since syphilis is readily treatable, participation in clinical trials for a vaccine would be “lower stakes”:

*“It sounds like syphilis is treated pretty easily too. In my mind, which is – this is maybe very erroneous thinking. It’s a lower stakes than – okay, if one were to contract syphilis, it can be treated pretty readily versus, with COVID, it seems more – that would be more of a – okay – a more higher risk.”* [P8]

Another interviewee noted that in terms of trial participation, the curability of syphilis is the key to their interest in participation since “*the fact that I could take an antibiotic and get rid of it, it would make me less anxious to do it. If we’d done that with COVID, okay, take this and that, but this is a whole different matter”* [P15].

### Interpersonal-level facilitator: trustworthy researchers

A prominent facilitator for potential trial participants was regarding the trustworthiness of the researchers. Trustworthiness was described in a variety of ways, including ensuring that trial participants are treated with respect. As one interviewee noted, it would be important for researchers to ensure that trial participants “*don’t feel like they’re not being judged in any way. That’s huge … that they’re respected. It’s so easy for people to look down on other people, and that never works … The trust factor is huge”* [P15]. Others noted the importance of ongoing communication in building trust between participants and researchers, which involves “*building up a relationship just by doing it and seeing each other more often and stuff like that through the visits … ’Cause the important thing is you’re working together in a sense … I’ve gotta be able to trust you to put this in my body to see, you know what I mean, to test it?”* [P21]. Multiple participants emphasised transparency and thorough explanation of procedures as essential to trust, which helps a person feel that “*you can believe what they say, and then you can really feel from them that they tellin’ you the truth”* [P27].

Having shared identities with trial participants – or at least an understanding of trial participants’ experiences – also was noted as an important factor in trustworthiness. As one interviewee explained, if “*[researchers] want to look at gay men, have another gay man talking to them. That would make you feel good, or have a woman talk to a woman, a Black person talk to a Black person, those kinda things to build that trust”* [P14] between trial participants and members of the research team. Another participant further explained that being knowledgeable about transgender health would be important for facilitating trust in the research relationship, noting that “*knowing – being knowing and accepting in regards to – ‘cause I’m trans. Knowing the health issues and just parameters and things that come with that is a sign of being a good doctor or a good researcher”* [P10].

### Institutional-level facilitator: trust in the research design/organisation

For some participants, the theme of trust as a facilitator extended beyond interpersonal-level factors to the institutional level, such that trust in the trial design or the organisation conducting the trial is of central importance in participation decisions. For example, an interviewee explained that interest in trial participation was contingent upon the organisation conducting the study:

*“I guess it would depend on who was doing the trial. If it were a public institution like a UNC Public Health or medical school versus the private sector, I think it … I’m sure that these are joined efforts, but I think I would trust a public health – if it were framed more like a public-health initiative versus big pharma.”* [P8]

For some interviewees, trust in research has grown through participation in previous studies and positive experiences with the way the research was organised:

*“Well, just because having done so many studies in the past, I actually just basically feel good being part of the research … I think it’s important that people participate, and I’m one of those who trusts the scientists [laughs], and I’ve been taken such good care of through these clinical trials that I always feel good about doing ‘em.”* [P13]

### Community-level facilitator: advancing the science/ending suffering

By far the most prominent facilitator of trial participation was the opportunity to contribute to advancing the science of syphilis prevention and ending the suffering caused by syphilis. Multiple interviewees explained the opportunity to be “*a part of history”* [P1] in contributing to a vaccine “*would make it really worth [participating]”* [P14]. Interviewees noted that participation in clinical trials is for the greater good, “*because that’s the only way we’re going to get [a vaccine] approved and find out how it works, and how safe and effective it is”* [P11]. Some interviewees specifically noted that their interest in trial participation was due to having watched the interview video which explained some basic statistics on the number of syphilis infections in the US and transmission pathways, including transmission of syphilis from pregnant people to their babies. As one participant noted, learning about the impact of syphilis “*motivates me to want to be in a trial and stop that because that’s … completely preventable. I’m just surprised that that’s still happening today. That was probably the most important part of the video for me, just hearing about the burden that it has on people”* [P19]. Participating in vaccine development could help eliminate the cycle of syphilis infection, testing, contact-tracing, and treatment:

*“ … if you get infected with syphilis, it’s automatically reported. If you test, it’s reported. You start getting those phone calls … It’s the time it takes to contact these people or make the contact. It’s the feeling of betrayal if you give up names, and that’s difficult, and so for me, the participation [in the trial] would be, let’s end all that, and you’re not gonna end that if you don’t have anybody that raises their hand and says ‘here I am’. I’ll do it 'cause if nobody does it, then all of your research, there’s nothing you can do.”* [P23]

### Policy-level facilitator: compensation

Several interviewees noted the importance of compensation. Some felt that it was difficult to put a true dollar value on the idea of compensation for the “total burden” [P2] of trial participation, as this could “*never cover the time and the energy and what happen to our human body when we been tested [on]”* [P5]. Nonetheless, interviewees noted that compensation was essential for participation in a vaccine clinical trial. Covering transportation costs, parking, meals, and accommodation if necessary were all mentioned by interviewees. Additionally, compensation for time and effort was noted as necessary:

*“If a drug company is eventually gonna make money off of something, they do need to give a financial benefit to the participants as well. I think it’s just – it’s fair and necessary. Then also just to cover the – it is time. You’re working, basically. You have to commute there and give hours and hours, and it is stressful to like, oh, I need to do a blood draw today. I think, for a lot of reasons, compensating participants is important.”* [P19]

### Policy-level facilitator: regulation of clinical trials

A final facilitator expressed by some interviewees was related to the ways that clinical trials are regulated – beyond the oversight of clinical trial researchers. For example, one participant was encouraged knowing that if a trial had been “*cleared … for a phase one, that there’s appropriate controls in that trial”* [P2]. Another participant noted that he would prefer to be in a later-stage clinical trial rather than a Phase 1 design because “*after it’s been tested and it’s on the final stages, you already been through the side effects with a lot of other people other than me. I wanna be in one that’s ready, FDA-approved, about to approved, it’s already sent, they just gotta sign [laughs]”* [P4].

### Considerations for community engagement

Participants also provided further insights to inform community engagement strategies. The majority noted that having community input on trials will be essential to establishing trust in the research and in the vaccine that emerges from the trials; as one participant explained, “*people like to know that your stuff is made locally, or that your product is locally, or that somebody from a local basis is on the research, or a local entity is on the research. I think that makes a great difference”* for community trust in the vaccine [P28]. In terms of which communities to involve in vaccine development, participants prioritised involvement of people who previously had syphilis as well as communities most impacted by syphilis and other health disparities, including Black/African American and LGBTQ communities. Some interviewees noted that sexually active young people should also be engaged.

Finally, participants suggested strategies for community engagement efforts on the topic of syphilis vaccine development. A summary of these suggested strategies is presented in Table 3.

**Table 3.** Summary of engagement strategies for syphilis vaccine research suggested by interviewees, organized by social ecological model levels

Strategy level	Engagement Strategy
**Interpersonal**	Pop-up Information booths/clinics
**Organizational**	Research open house Information integrated into public schools
**Community**	Town halls/public forums Announcements in churches Collaboration with existing community organizations
**Policy**	Flyer campaigns Paid public advertisements

Collectively, all suggestions focused on community research literacy and outreach as a first step in the engagement process, with participants emphasising the need to inform communities about both syphilis and syphilis vaccine trials. One participant emphasised the importance of community-focused informational campaigns prior to the start of any clinical trials:

*“I think before you start recruiting, I think going out and educating about syphilis, it will be the first – educating about syphilis, educating about vac – kinda like this video. Have education sessions, not a recruiting session, just education sessions. I think that will help a long way with recruiting in the future, starting off.”* [P30]

However, several participants also noted the need for researchers to be visible within the community and to reassure community members that the research is being conducted by people who are passionate and genuinely want to make a difference; this could be done by “*holding a town hall for example. Like, “We’re here doing this research. This is who we are. Again, we’re humans. We want to help humans. This is how we’re doing it’”* [P14]. One participant explained that community engagement goes together with researchers making themselves more visible in communities:

*“I just think visibility is a big thing. If people are seeing you in the community, and name recognition and stuff, where everybody knows UNC. I know you, but I don’t see you in my hood. I don’t see you in my community. I know where you are, but do you know where I am? I guess what I’m saying is people know when you have a genuine interest, and people know when you just want something.”* [P28]

## Discussion

This qualitative study offers a starting point for understanding the perceptions of priority populations regarding participation in syphilis vaccine trials and what barriers and facilitators they see as impacting trial participation. Much previous work in studies of vaccine willingness has focused on perceptions of receiving an STI vaccine;^30^ however, trial participation *per se* is a unique concept that should be considered separately from vaccination. Our study explored the perceptions of key populations from a southeastern US state in which concerns regarding the history of syphilis research were expressed by several participants. Despite these concerns, we found that overall interest in syphilis vaccination and trial participation was high. These findings have important global health implications for addressing the burden of syphilis infection by offering an initial positive indication that our study populations would be willing to participate in vaccine discovery trials, and that a syphilis vaccine would be an acceptable and indeed desirable option for syphilis prevention. Given the disproportionate impact of syphilis on marginalised populations in the US^21,31^ and globally,^32^ our findings lend further support to the importance of developing a syphilis vaccine and to ensuring access to vaccination among populations experiencing higher rates of syphilis infection once a vaccine is available.

Our findings regarding trial participation barriers and facilitators have important implications for trial design. Mistrust and fear of physical harm were identified as potential barriers to syphilis vaccine trial participation. Fear of side effects, concerns about vaccine efficacy, and community mistrust have similarly been reported in studies of willingness to participate in HIV vaccine trials.^33–36^ In terms of facilitators, altruism has been shown to be a primary motivator in preventative vaccine trial participation.^37^ Thus our findings suggest that syphilis vaccine trials will similarly require enhanced transparency, thorough communication with trial participants, and cultural sensitivity in developing and delivering trial information, as has been observed in other forms of vaccine research.^38^ Our findings further suggest that cultural understanding of concerns among racial/ethnic and sexual/gender minoritised populations would be of central importance. To maximise the impact of these findings for global public health, further investigation is needed to understand whether and how concerns differ across international settings to effectively tailor communication about trial processes in ways that are culturally acceptable and appropriate.

Our data suggest that individual perceptions of unethical research may influence syphilis vaccine trial participation. The Tuskegee Syphilis Study was raised by multiple interviewees without any prompting. Tuskegee has previously been shown to be a significant barrier to medical research participation among African Americans.^13^ Our findings demonstrate an ongoing need for syphilis vaccine researchers to grapple with the lasting impact that the history of syphilis research may have on participation in trials by African Americans.^14,39^ Others have noted the impact of Tuskegee on HIV vaccine trial recruitment, with potential solutions including increased community outreach, transparency in research processes, and an emphasis on trial participation as a way to ameliorate health disparities.^40^ We can also learn from recent efforts to improve community mistrust in medical research amidst the COVID-19 pandemic through the use of trained community engagement coordinators drawn from the communities one seeks to reach (e.g. minoritised populations).^41^ These strategies may similarly help to address our finding of community mistrust as a barrier to syphilis vaccine research.

Several participants noted that syphilis vaccine trial participation would be a relatively “low stake” scenario, given the curability of syphilis should the vaccine not work to protect from syphilis infection. Improving community understanding of complications from untreated syphilis (like ocular syphilis or tertiary syphilis), risks from treatment failures (like neurosyphilis) and transmission (to partners and infants) may thus be important elements in education and fostering community buy-in for syphilis vaccine trials, as a more accurate understanding of the risks of syphilis could aid in leveraging our observed trial participation facilitator of advancing research to end suffering. There is some evidence to suggest that community health literacy may similarly help to facilitate participation in gonorrhoea vaccine trials.^42^ Given that our participants’ strategies for community engagement largely focused on educational outreach, practical strategies to improve community understanding about syphilis should be a focus of future engagement efforts. It may also be important to consider the impact of age on developing effective and tailored engagement strategies; given that slightly over half of our participants were age 45 and up, investigating the most effective ways of informing both younger and older populations on the topic of syphilis vaccine research could result in greater vaccine trial participation interest.

There are some important limitations to this study. First, recruitment was limited to a convenience sample of interviewees from central North Carolina, and due to our use of a self-referral recruitment strategy, the number of participants referred to our study via each of our specific recruitment sites is unknown. However, this region of North Carolina houses an experienced HIV clinical research site (UNC Chapel Hill) which would likely be involved in early syphilis vaccine trials. Second, interviewees were asked to consider the question of hypothetical participation in syphilis vaccine clinical trials. Prior research has shown the potential for large discrepancies between reported willingness to participate in future vaccine trials and true enrolment.^43^ However, at this point in time, all syphilis vaccine research in human subjects is hypothetical, and evidence from HIV vaccine research has demonstrated the importance of early engagement.^44–46^ Our study team included world-leading syphilologists conducting vaccine work, which helped to ensure realistic explanation of future vaccine trials. Additionally, our interviewee sample was highly experienced with participation in previous clinical research. This may have impacted participants’ perceptions of barriers and facilitators of trial participation, as well as biased their propensity towards willingness to participate, given a potentially much higher level of understanding of clinical research processes among our participants. However, this same previous research experience is what may make these participants well-positioned to reflect on decision-making when considering trial participation, as well as potentially more likely to pursue syphilis vaccine trial participation opportunities in the future. We therefore consider it a strength of our study that most of our interviewees had prior research experience, positioning them to inform us how we could address their fears, leverage their interests/priorities, and reach their communities (MSM, PLWH).

Our findings suggest that a priority area for syphilis vaccine researchers would be to partner with community engagement experts to address the barriers identified in our study and leverage facilitators to encourage trial participation. Adopting an explicitly anti-racist framework for conducting medical research is essential,^47^ particularly given the lasting impact of Tuskegee on the disproportionate underrepresentation of African Americans in clinical studies.^39^ Future work is also needed to obtain the perspectives of populations in other locations where syphilis clinical trials are likely to be held, including outside of the US, given the crucial need for community consultation.^44–46^ In addition, research is needed to understand whether and how the barriers and facilitators identified in the current study impact trial participation among populations disproportionately impacted by syphilis worldwide.

## Conclusions

Priority populations identified a wide range of barriers and facilitators that may impact syphilis vaccine trial participation, with factors ranging across the personal, interpersonal, organisational, community, and policy levels. These findings will help syphilis vaccine researchers anticipate challenges in trial participation. They also point to important lessons for enhancing community engagement, designing and conducting trials, and involving priority groups in the community consultation processes. Research will be needed to identify effective ways of designing engagement strategies to address the barriers of greatest concern to communities of interest, given the history of syphilis research, while maximising the potential impact of participation facilitators.

## Supplementary Material

Supplemental Material 1. Semi-structured interview guide.

## Data statement

De-identified data are available from the authors upon reasonable request.
