# Supplementary material for: Barriers and facilitators of participation in syphilis vaccine trials: a qualitative analysis to inform trial design and community engagement in the United States
Source: Sex Reprod Health Matters. 2025 Mar 7;32(1):2473199. doi: 10.1080/26410397.2025.2473199 (PMC12051577; doi:10.1080/26410397.2025.2473199)
Supplement: Supplemental Material 1. Semi-structured interview guide. [file ZRHM_A_2473199_SM2241.docx]

**Supplemental Material 1. Semi-structured interview guide.**

**Interview Guide:**

**Barriers and Facilitators of Syphilis Vaccine Trial Participation**

1. I’d like to start us off by first talking a little bit about vaccines. A vaccine is a medical product that helps to protect against certain diseases.
   1. One vaccine that has been on everyone’s minds lately is the COVID-19 vaccine. What are your thoughts on the COVID-19 vaccine?
      1. What do you think about getting the COVID-19 vaccine? Have you received it yet? (If no: Do you want to get it? Why or why not?)
   2. One vaccine that is received as a teen or young adult is the vaccine against human papilloma virus, or HPV. HPV is a very common sexually transmitted infection. Were you ever offered the HPV vaccine?
      1. If yes: Can you tell me more about that – did you end up getting the vaccine? (If Yes: when did you get the vaccine? What was the experience like? If No: can you tell me more about that – why didn’t you get the vaccine?)
      2. If no: if you were eligible for this vaccine, would you be interested in getting it? Why or why not?
   3. Are there any other vaccines you have had as an adult?
      1. Are there any vaccines you have missed, or that were offered to you but you chose not to get?
      2. Do you get the annual flu shot? Why or why not?
2. Next I’d like to talk about syphilis:
   1. Can you tell me a bit about what you know about syphilis? Prompts: what is it? How is it transmitted? How is it treated? How can it be prevented?
   2. Who are the people that get syphilis? Are there groups of people who are at greater risk of syphilis? If so, who?
   3. What do you think about when you hear the term “syphilis research”?
   4. What kinds of things do you think researchers are interesting in studying about syphilis?
3. I’d like to hear about your previous experiences with medical research. Have you ever been in a medical research study before?
   1. If yes: What was the study about? Can you tell me about what your experience was like?
   2. If no: Have you ever applied to be in medical research study? Would there be any medical research that you might be interested to participate in in the future?
4. Right now, scientists are conducting medical research to try to develop a vaccine that would prevent a person from being infected with syphilis. What do you think about the idea of a syphilis vaccine?
   1. Prompt: do you think a syphilis vaccine would be important or useful? Why or why not?
   2. Who needs a syphilis vaccine the most?
   3. Who would be the most important groups of people to focus on when developing a syphilis vaccine? Prompt: are there groups that researchers should especially try to get involved in vaccine studies?

*Before we go to our next questions, I’m going to show you a brief video about syphilis vaccine research. This will take about 5 minutes to view, and it will explain what syphilis vaccine studies will look like and what kinds of processes would be involved in participating in these studies. So let’s watch it together and then I will have more questions for you.*

*[Interviewer plays an animated video.]*

1. Let’s talk about your thoughts and impressions of syphilis vaccine research. What do you think of the idea of participating in such a study?
   1. Prompt: Is participating in early stage syphilis vaccine clinical trial something you think you’d be interested in, once studies start looking for volunteers to participate?
      1. If yes: Why? Can you tell me more about that?
      2. If no: Why not? Are there things that would make you more likely to participate?
2. Imagine you are a participant in a syphilis vaccine study:
   1. What would be some of the things you’d be worried about?
   2. What would be some of the things you’d be looking forward to or excited about as a study participant?
3. Participating in a syphilis vaccine study would likely involve committing to participating in the study over a long period of time, likely over the course of a year or more. How would you feel about this? Are there things researchers do to make participating in a lengthy study more appealing?
4. Syphilis vaccine studies would pay people to participate. What do you think about compensating people to participate in a syphilis vaccine trial?
   1. How important would compensation for trial participation be for you?
   2. What would fair compensation look like to you?
5. I’d like to talk about the relationship between researchers and study participants. For a syphilis vaccine study:
   1. What would a good relationship between researchers and study participants look like?
      1. What could researchers do to build good relationships with study participants?
      2. How could researchers earn participants’ trust?
   2. What would be some things that could harm relationships with study participants?
   3. What about the relationship between researchers and the community where a syphilis vaccine study is being conducted – what would a good relationship look like?
   4. What are some things that could harm the relationship between researchers and the community where a syphilis vaccine study is being conducted?
   5. What about community involvement in helping to organize and provide feedback on the research: how important do you think this would be for syphilis vaccine research?
      1. what difference could this make for trust in a syphilis vaccine?
      2. Would you trust a vaccine more if study participants and other from the community were involved in helping to organize the research? Why or why not?
   6. Are there groups of people that it would be more important for researchers to consult with when developing a syphilis vaccine? Who?
      1. As research progresses towards a syphilis vaccine, who should researchers be reporting their findings to? Would you be interested in learning about how the research is progressing? Why or why not?
6. Let’s talk about how people participating in a syphilis vaccine study might be seen by others. Thinking about your own close networks of friends and family:
   1. If you were participating in a syphilis vaccine study, how would you feel about telling people about this? [Prompt: would this be something you would talk about with your sexual partner(s), friends or family? What about people in your community, like your neighbors?]
   2. How do you think your partner(s), friends and family would feel if they knew you were a participant in a syphilis vaccine study?
7. Finally, I’d like to ask you: if researchers were successful in developing a syphilis vaccine, and it became available to the public, would you want to be vaccinated? Why or why not?
